# Quality of reports of investigations of research integrity by academic institutions

**DOI:** 10.1186/s41073-019-0062-x

**Published:** 2019-02-19

**Authors:** Andrew Grey, Mark Bolland, Greg Gamble, Alison Avenell

**Affiliations:** 1 Department of Medicine, University of Auckland, Private Bag 92019, Auckland, 1142 New Zealand; 2 Health Services Research Unit, University of Aberdeen, Aberdeen, UK

**Keywords:** Research integrity, Institution, Misconduct, Investigation

## Abstract

**Background:**

Academic institutions play important roles in protecting and preserving research integrity. Concerns have been expressed about the objectivity, adequacy and transparency of institutional investigations of potentially compromised research integrity. We assessed the reports provided to us of investigations by three academic institutions of a large body of overlapping research with potentially compromised integrity.

**Methods:**

In 2017, we raised concerns with four academic institutions about the integrity of > 200 publications co-authored by an overlapping set of researchers. Each institution initiated an investigation. By November 2018, three had reported to us the results of their investigations, but only one report was publicly available. Two investigators independently assessed each available report using a published 26-item checklist designed to determine the quality and adequacy of institutional investigations of research integrity. Each assessor recorded additional comments ad hoc.

**Results:**

Concerns raised with the institutions were overlapping, wide-ranging and included those which were both general and publication-specific. The number of potentially affected publications at individual institutions ranged from 34 to 200. The duration of investigation by the three institutions which provided reports was 8–17 months. These investigations covered 14%, 15% and 77%, respectively, of potentially affected publications. Between-assessor agreement using the quality checklist was 0.68, 0.72 and 0.65 for each report. Only 4/78 individual checklist items were addressed adequately: a further 14 could not be assessed. Each report was graded inadequate overall. Reports failed to address publication-specific concerns and focussed more strongly on determining research misconduct than evaluating the integrity of publications.

**Conclusions:**

Our analyses identify important deficiencies in the quality and reporting of institutional investigation of concerns about the integrity of a large body of research reported by an overlapping set of researchers. They reinforce disquiet about the ability of institutions to rigorously and objectively oversee integrity of research conducted by their own employees.

## Background

The integrity of the biomedical literature is fundamentally important in ensuring the efficient development of effective and safe clinical practices. Protecting research integrity is the responsibility of several protagonists, including individual researchers, peers and coinvestigators within research groups, funding bodies, regulatory bodies, journals, publishers, and institutions that employ research staff and administer research activities.

Academic institutions can play essential roles in the protection of research integrity, by providing supportive environments and demonstrating best practice [1]. Because institutions have direct access to researchers they employ and are frequently directly involved in research administration, they can both investigate whether research is reliable and determine whether research misconduct has occurred. Consequently, institutions are frequently involved in cases of suspected compromise of research integrity. However, concerns exist about the reliability, consistency, transparency and completeness of institutional investigations [2, 3]. Suggestions to address these concerns include quality checklists for institutional investigations and their reports, and independent peer review and publication of reports [1].

Published research on institutional investigations of research integrity is infrequent. Between 2013 and 2016, we raised wide-ranging concerns about the integrity of 33 clinical trial reports from a research group in Japan [4]. By early 2017, as a result of investigations initiated by affected journals, 12 retractions had ensued, for fabrication, plagiarism, authorship misconduct and unresolved concerns about data integrity. Contemporaneously, we were concerned about the integrity of the larger body of non-randomised and preclinical research reported by the same group, but to our knowledge, no systematic investigation of this work was being undertaken by publishers of affected journals, and no institutional investigation had been undertaken or was underway. Beginning in March 2017, we raised wide-ranging concerns about the integrity of > 200 publications with the three Japanese and one US universities of affiliation of the two leaders of the research group. Here, we assess the reports of the institutional investigations conducted in response to our concerns.

## Methods

We searched the website of each affected institution to identify staff with responsibility for oversight of research integrity and communicated by email. Our initial email to each institution outlined the context of compromised integrity and retractions of work published by employees, the publicly available reasons for existing retractions, the absence of scrutiny of non-randomised and preclinical research by those employees, and a suggestion that the institution should assess the integrity of all potentially compromised publications co-authored by the employees. Detailed concerns about specific publications were communicated to three of four institutions at either first contact or in subsequent emails. Each institution was appraised of the involvement of employees of the other institutions and that we had raised concerns with the other institutions. Each institution was also made aware that we had sent detailed concerns to the national organisations responsible for research integrity oversight [Office of Research Integrity (ORI) in the USA; Ministry of Education, Culture, Sports, Science and Technology (MEXT) in Japan].

We defined a potentially affected publication as any paper authored by the primary respondent with at least one co-author affiliated to the institution. We defined affected employees as those who co-authored > 5 publications with the primary respondent.

Each institution initiated an investigation. By 3 December 2018, three had reported their results to us. Only one report is publicly available [5]. Using a recently published checklist that was designed by a panel of experts in the evaluation of research integrity [1], two of us (AG, MB) independently assessed the quality of each report and resolved differences by consensus. The checklist includes an overall assessment, with four categories that include ‘acceptable as is’, ‘minor revisions needed’, ‘major revisions needed’ and ‘report is not acceptable’.

## Results

During correspondence with the institutions, we learned that two institutions had undertaken investigations prior to receiving our concerns, but neither was publicly announced or reported. Institution 1 undertook a preliminary investigation 11/16–1/17, but commenced a full investigation in March 2017. Institution 2 undertook an investigation 3/16–1/17 which found ‘improper scientific (authorship) conduct’ but did not lead to correction of any affected publications.

The ORI did not acknowledge receipt of emails sent in October and November 2017 outlining concerns about research conducted at the US institution. Our emails to the MEXT in November and December 2017 reporting the concerns about research conducted at the Japanese institutions, including ones written by a Japanese colleague, either failed to elicit a response or generated brief unhelpful replies, promising a response that has not yet materialised.

Table 1 sets out the timelines of our notification of the affected institutions, the nature of the concerns raised and the scope of the concerns. The concerns raised overlapped between institutions and with those raised in regard to the clinical trials which were the focus of a published systematic review [4]. At each institution, ≥ 2 employees (range 2–6) were co-authors on ≥ 5 potentially affected papers. The number of potentially affected publications at individual institutions ranged from 34 to 200.

**Table 1:** Notification, nature and scope of concerns about research integrity raised with four academic institutions

	Institution			
	1	2	3	4
Concerns				
Date(s) concerns raised by our group	14 March 2017	14 March 2017 15 May 2017 11 October 2017 18 November 2017	14 March 2017 18 November 2017	11 October 2017 24 November 2017 3 May 2018
Types of concern	Fabrication Falsification Plagiarism Authorship misconduct Study conduct and governance irregularities Implausible productivity Implausible data Data errors	Fabrication Falsification Plagiarism Duplicate reporting Authorship misconduct Study conduct and governance irregularities Implausible productivity Impossible data Implausible data Data errors Funding unclear	Fabrication Falsification Plagiarism Duplicate reporting Authorship misconduct Study conduct and governance irregularities Implausible productivity Impossible data Implausible data Data errors Funding unclear	Fabrication Plagiarism Duplicate reporting Authorship misconduct Study conduct and governance irregularities Impossible data Data errors Funding unclear
Detailed concerns raised about specific publications	N	Y	Y	Y
Employees affected,^a^ *N*	4	4	6	2
Publications potentially affected,^b^ *N*	49	200	68	34

^a^Co-author > 5 publications with primary respondent

^b^Papers authored by primary respondent with at least one co-author affiliated to the institution

Table 2 summarises the processes and outcomes of the investigations by the three reporting institutions, which took 8–17 months. Institution 3 has not reported the outcomes of its investigation to us or, to our knowledge, publicly. Institution 2 focused on one employee—it was unclear how many were investigated by institutions 1 and 4. Institutions 1, 2 and 4 investigated 38/49, 7/200 and 5/34 potentially affected papers, respectively. In subsequent correspondence, institution 2 indicated it was investigating non-clinical research by its employee.

**Table 2:** Scope and outcomes of investigations undertaken by four academic institutions

	Institution			
	1^a^	2^c^	3	4
Investigations by academic institutions				
Scope	38 papers, first author primary respondent	7 papers, first author primary respondent	Uncertain	5 papers, first author primary respondent
Employees investigated, *N*	Uncertain	1	Uncertain	Uncertain
Date of report	17 November 2017	23 August 2018	Not available	30 May 2018
Decisions	Research misconduct primary respondent No research misconduct other employees associated with investigated papers	No misconduct	Not available	Duplicate reporting present ‘Concerns about integrity of research well-justified’
Recommendations	Retraction of 14 papers, 7 of which were already retracted. Remaining 24 papers, no clear evidence of fraud	Retraction of 2 papers, to be actioned by primary respondent. Noted 1 existing retraction, overlooked another.	Not available	No action
Report publicly available	Yes^b^	No	Not applicable	No
Subsequent response from our group	Raised specific concerns about 18 papers for which the investigation could not determine integrity	Reiterated concerns about > 100 papers	Not applicable	Concerns not addressed or resolved for any publication
Current status	Further investigation commenced	Uncertain	Ongoing	Further investigation commenced

^a^Preliminary investigation November 2016–January 2017, full investigation commenced March 2017

^b^Japanese language only

^c^Earlier investigation March 2016–January 2017

Table 3 shows our assessment of the institutions’ reports. Assessors agreed on 21, 21 and 20 of 26 items in each report (kappa coefficients 0.68, 0.72 and 0.65, respectively). Consensus resolved the minor differences that occurred because of ambiguity in the questions in the Evidence section. Overall, each report was considered unacceptable by both assessors. In the respective reports, only 3/26, 1/26 and 0/26 individual checklist items were addressed adequately: a further 5/26, 5/26 and 4/26 items could not be assessed. Common deficiencies were widespread and occurred in each domain of the checklist, including the presentation of concerns/allegations, scope of investigation, descriptions of the investigating committee, its resources and conflicts, description of evidence considered/not considered, clarity of presentation of findings and conclusions, and support of conclusions by the evidence.

**Table 3:** Quality assessment of institutions’ reports on the investigation of research integrity

	Institution^a^		
	1	2	4
Scope			
Includes executive summary	No	No	No
Clear and understandable	In part	In part	In part
Allegations clearly presented	In part	No	No
Charge to committee clearly described	No	No	No
Scope sufficient to address scientific integrity issues	No	No	No
Investigative committee			
Appropriately constituted	Cannot assess	Cannot assess	Cannot assess
Any external members	*Yes*	*Yes*	Cannot assess
Potential conflicts of interest reviewed	No	No	No
Report indicates standards of due process and confidentiality followed	No	No	No
Respondent had opportunity to identify conflicts	Cannot assess	Cannot assess	Cannot assess
Any concern that committee lacked expertise and resources	Yes	Yes	Yes
Evidence			
Report indicates evidence sequestered and protected	No	No	No
Description of evidence considered	In part	In part	In part
Respondent offered opportunity to respond	Cannot assess	Cannot assess	Cannot assess
Committee considered and addressed whether important evidence was unavailable	In part	Cannot assess	In part
Explanation for failure to review seemingly pertinent evidence	In part	No	No
Need for further evidence or additional analysis	Yes	Yes	Yes
List of individuals interviewed	No	No	No
Should others have been interviewed	Cannot assess	Cannot assess	Yes
Additional questions that should have been asked or evidence examined to reach a supportable conclusion	Yes	Yes	Yes
Conclusion			
Report clearly states findings	*Yes*	In part	In part
Report clearly states conclusions	*Yes*	In part	In part
Evidence fully support conclusions	Cannot assess	No	No
Articulates and applies institutional policies	No	No	No
Recommendations clear and supported by report	No	No	No
Report describes and addresses requirements of external sponsors’ regulations	No	No	No
Overall assessment	Not acceptable	Not acceptable	Not acceptable

Derived from Gunsalus et al. Components considered to be adequately addressed are in italic type

^a^Institution 3 has not reported the results of its investigation

Specific and detailed concerns were raised about potentially affected publications co-authored by employees at institutions 2, 3 and 4. These concerns were wide-ranging, including those about research governance, authorship misconduct, implausible productivity and study conduct, duplicate results reporting, impossible or implausible data, and data errors (Table 1). Institution 3 has not disclosed the outcomes of its investigation, but correspondence from institutions 2 and 4 that reported to us the results of the investigations did not address any of the specific concerns about individual publications. In none of the reports was it apparent that any institution had collaborated with any of the other institutions. Each assessor of the reports considered that the investigations focussed on potential misconduct, restricted in definition to fabrication, falsification and plagiarism, rather than the integrity of all potentially affected publications. For example, the investigation committee of institution 1 reserved judgement on 24/38 papers because it ‘did not obtain clear evidence of fraud for these publications.’ When we subsequently communicated specific concerns about a wide range of aspects of the integrity of 18 of these 24 papers (concerns listed in Table 1), institution 1 initiated further investigation.

After receiving each report, we raised or reiterated many unresolved concerns with each institution, 2 of which then commenced additional investigations.

## Discussion

Institutional investigations are important in protecting and restoring research integrity, but assessment of the adequacy of the processes undertaken and the quality of the ensuing reports is uncommon, in part because they are often opaque and/or inaccessible, respectively [1, 6]. Only one of the three reports we analysed is publicly available, so key stakeholders such as the wider research community, health practitioners and patients are unaware of the existence of two of the investigations, let alone their outcomes. In two cases, we do not know whether the reports have been shared with regulatory bodies. The investigations failed to assess the majority of publications about which concerns were raised and, when detailed concerns were raised about specific publications, did not address them. The reports of the investigations were inadequate in almost every respect, as judged by scoring using a quality checklist. They contrast markedly with a higher quality report of an institutional investigation of a different case [7]. Consequently, it is impossible to be confident that rigorous, comprehensive and objective investigations were undertaken. The upshot is that the most important outcome, the validity and reliability of the published research, remains uncertain.

To our knowledge, few analyses of institutional investigations of research integrity have been undertaken, and we are unware of analyses of investigations by multiple institutions of a common set of concerns. The Retraction Watch website reports the scarcity of information from institutional investigations, which, when it is available, often became so via legal proceedings or public records requests [8]. Occasionally, high-profile cases have attracted editorial commentary, which has been both complimentary [3] and critical [6, 9] of the institutions’ performances. Overall, there is disquiet about institutional investigative processes, because of concerns about conflicts of interest, inadequate oversight and too little transparency [1].

We found that that institutional investigations failed to address all potentially affected papers and to address detailed concerns about individual publications. The latter observation is similar to that in an analysis of journal and publisher responses to such concerns over research published by the same group of researchers (data in submission). In that analysis, none of the 16 decision emails from affected journals responded to all of the specific concerns that had been raised about the relevant publication. We cannot determine the reason for these findings. It could be that the institutional investigations decided to focus on a subset of potentially affected papers, did not seek the evidence required to address all the concerns, or that evidence was not available or provided, or that the investigation was conducted and/or the report was written without consideration of those specific concerns. The considerable scope of the concerns and the involvement of more than one institution and more than one country might have influenced the quality of the investigations undertaken. The lack of evidence in the reports of communication between affected institutions also aligns with the very limited communications that appeared to take place, at least to our knowledge, between affected journals after concerns were raised (data in submission).

Our assessment of two of the three available investigation reports suggested a much stronger focus on the determination of misconduct, restricted in definition to fabrication, falsification or plagiarism, than the evaluation of research integrity; the third report was too vague to assess in this regard. This observation is supported by the failure of the investigations to address specific concerns about the integrity of individual publications which included those about research governance, authorship misconduct, implausible productivity and study conduct, duplicate results reporting, impossible or implausible data, and data errors. We think this an important observation. We suggest that the integrity of published research should be the first priority of any investigation of concerns, as integrity can be importantly compromised without evidence of misconduct, particularly if narrow definitions of the latter are applied. Conflating research integrity and misconduct may inhibit achieving the critical goal of protecting the integrity of the published literature.

## Conclusions

Our analyses identify important deficiencies in the quality and reporting of institutional investigation of concerns about research integrity. They reinforce disquiet about the ability of institutions to rigorously and objectively oversee the integrity of research conducted by their own employees and the lack of regulatory oversight. A possible solution is the establishment of more efficient and adequately resourced independent organisations with the authority and expertise to undertake and report investigations, and implement recommendations, including those which span multiple institutions and countries.
